# The Hospital Nursing Supplement

**Published:** 1893-06-24

**Authors:** 


*The Hospital*, June 24, 1893. *Extra Supplement.*

### "The Hospital" Nursing Mirror.

Being the Extra Nursing Supplement of "The Hospital" Newspaper.

[Contributions for this Supplement should be addressed to the Editor, The Hospital, 428, Strand, London, W.C., and should have the word "Nursing" plainly written in left-hand top corner of the envelope.]

### News from the Nursing World.

### A ROYAL VISIT TO CLERKENWELL.

On Saturday, the 24th inst., the Prince of Wales will unveil the memorial of the late Duke of Clarence at the Hospital of St. John of Jerusalem, at Clerkenwell. The Queen is Sovereign Head and Patron, the Prince of Wales Grand Prior, and the late Duke was the first Superior of the Order since its incorporation. It is expected that some of the members of the Royal family will accompany the Prince on Saturday, and that he will present the medals and certificates which the Order has awarded during the past year for gallantry in saving life on land.

### THE ROYAL WEDDING.

There is a great similarity in the letters from Pension Fund Nurses this week, for they all contain suggestions for the Wedding Day. Everybody so far seems to think that a stand from which a sight could be obtained of the bride and bridegroom and the Royal procession would be the greatest possible treat. But of course this cannot be done without a small cost to each nurse. Those who are aware that their duties will keep them from personally sharing in the general holiday urge that their sister nurses should, if possible, be amongst the spectators. We hope to receive communications from nurses who have not yet written as soon as posssible, then we shall learn what the majority desire to do on the Royal Wedding Day. Post cards can be sent to the Manager of The Hospital, 428, Strand, and they need only bear the words "Procession" or "Picnic," with each nurse's name and policy number.

### THE NURSE DOLLS.

Some charming nursing uniforms have reached us, and each parcel of dolls has been acknowledged to the kind donors. One lady has written a friendly letter, in which she says, "although the name of this hospital is not included in the lists you published of uniforms you desired to have added to the collection of nurse dolls, my nurses, justly proud of their uniform, were anxious to be represented. They felt quite slighted by being omitted." We trust that no nurses will really imagine any slight or disparagement of their special uniform is conveyed by the omission of a hospital's name. It was quite impossible to make an exhaustive list, and we are much pleased to find nurses have willingly dressed dolls for the collection without being specially asked.

### OUR HOLIDAYS.

Nurses who are having their holidays during these hot weeks are to be congratulated, especially if they have secured congenial and pleasant surroundings. If the enjcyment of holidays consists in their having been earned by previous hard work, surely the leisure time of hospital nurses should be especially agreeable. Moreover, it is good to think of the number of institutions which are now ableto grant to each member of their nursing staff a reasonable amount of recreation during the year. Pleasant holidays are always too short, bu we hope all our readers will be able to secure in the course of the summer such change and rest as will send them back refreshed and invigorated to their daily work.

### "IN HOSPITAL."

Many paragraphs in the lay press deal with hospitals as they are not, as they never have been, and never will be. The writers have neither personal knowledge nor yet sufficient imagination, to treat of the hospital world as it really is, yet that after all is its most intensely interesting aspect. However, in the *Westminster Gazette* of 9th inst., there was an article headed "In Hospital," which is the accurate record of real life within the wards. No nurse could fail to enjoy such truthful observations as these. "In the education of a city it plays a part such as no Board school can ever fill. . . . Hospital life may also be compared to a return to Nature. Here lies the unsophisticated man, reduced to the primal need, the effort to continue alive." "Social and educational distinctions vanish in the overwhelming importance of the physical, and a pot-boy with serious complications excites greater interest than a minor poet whose case is minor too." The article is too good to be merely quoted from, but should be read by all who appreciate excellent writing on excellent subjects.

### DISCOURTESY TO A MATRON.

Judging from the report of an inquest at Northwich the other day, given in the *Liverpool Post*, the coroner appears to have treated with extraordinary discourtesy the Matron of the Victoria Infirmary. A patient who had been run over died from the effects of the accident, and an inquest was held at a public-house. In the course of the inquiry the coroner decided to summon the nurse of the ward. It appeared that neither she nor the Matron had any previous warning that they might be required to attend, and the verbal messages of the coroner did not receive the immediate attention he expected. Thereupon he made some very discourteous remarks, coupled with threats, and the infirmary doctor's assertion that he "was with the deceased when he died, and that the regulation of the hospital is that the nurse is not allowed to leave the infirmary," was received with laughter. Where the joke lay, however, does not exactly appear. We imagine the irate coroner and his facetious companions would at any time show small mercy to a nurse whose unauthorised absence from her ward was productive of evil consequences to her patients. Certainly the nurse's message to the effect that she could not leave the infirmary until she had seen the doctor was a perfectly right one. The subsequent threats of the coroner that she should be taken "into custody for contempt of my court," were uncalled for. The Matron eventually came forward herself, and managed to take a dignified position under most adverse and exceptional circumstances. This is by no means the first time that nurses have been rudely treated by those who ought to set a better example. If all papers followed the example of the *Liverpool Post* in giving publicity to such facts, they would soon cease to exist.

### SEA BREEZES.

A short residence by the sea does so much to complete the cure of sick children, that nurses hail with delight the establishment of every additional convalescent home. The medical staff of the Pendlebury Children's Hospital have taken a furnished cottage at St. Anne's-on-Sea in connection with their convalescent fund. This will enable a number of little patients to enjoy the health-giving sea breezes, which indeed bring new life to young convalescents.

### AN APPROPRIATE SUGGESTION.

Fever nurses, especially those engaged in the care of small-pox patients, deserve every consideration. To a certain extent they are shunned by their neighbours and friends, and are thus deprived of many of the diversions which fall to the lot of their sisters working in other branches of the profession. At Bradford the Vicar of Holy Trinity and the medical officer at the Fever Hospital seem entirely to realise the monotony of the lives of the small-pox nurses, and the former proposes that a fund should be raised to give them some country drives. Presumably two at least could be off duty together, and the possession of a pony carriage would put pleasant possibilities before all in turn. Busy nurses can never be dull, but they are often too tired to face the fatigue of the long walk which would eventuate in the country scenes longed for.

### NEGLECTED PAUPERS.

Some curious revelations as to the attendance on the sick paupers at Wolverhampton have recently been published. In the half-year ending last Michaelmas 699 patients were treated in the Workhouse Infirmary One male and one female nurse were paid to take charge of these sick persons, the title "nurse" being a merely formal one, as the people thus designated had neither of them received any special training for their duties. One Guardian made an assertion easily understood under such circumstances, that "on both sides of the house afflicted persons lay in a miserable state of neglect for hours because there was no one to look after them." The two responsible but untrained attendants have been assisted by pauper nurses, some of whom can neither read nor write, yet they have been trusted to administer medicines, &c. The statements of Dr. Totherick must not be ignored, and we cannot wonder at his saying that "he felt paralysed by finding that there were seven Guardians who could vote for shelving so important a question." The proposed innovation was sufficiently modest, as it consisted in a proposal to add two nurses to the staff. An excellent letter, signed "T. S. Drummond," appeared in the *Wolverhampton. Express*, and we agree with the writer that it is "the desire of the ratepayers that those of their neighbours who through misfortune have to spend the closing days of a hard life in the workhouse should have at least reasonable care in the days of sickness. The present state of things cannot be defended on economical grounds. Inefficiency is never economical—efficiency need not be extravagant." If other ratepayers recognise these truths the seven Guardians will not long be allowed to countenance the existing evils.

### ANOTHER QUEEN'S NURSE IN WALES.

The Llanelly District Nursing Association has a capital report sheet, and shows a balance in hand. Last November the Association became affiliated with the Welsh Branch of the Q.V.J.I., and secured the services of Miss Hosking, who is a Queen's Nurse. The report gives the following gratifying testimony to her ability: "Since she has been here she has proved herself most efficient and satisfactory in every way, both to the medical gentlemen who have employed her, and also to the patients."

### WHAT'S IN A NAME?

The Superintendent and Matron of the Convalescent Hospital, Lock-head, have resigned, and the Board of the Royal Infirmary, Aberdeen, have selected their successors. Only eleven candidates applied for the appointment, for which a salary of £45 per annum with residence and maintenance was offered. The Convalescent Hospital Committee reported that they had carefully considered the matter, and had decided on Mr. and Mrs. Charles, who for eleven years acted as porter and attendant at Oldmachar Poorhouse. We hope the name "Convalescent Hospital" is not accurately descriptive of the institution, otherwise it is reasonable to suppose that its inmates need the care of properly trained persons.

### AMERICAN TRAINING SCHOOLS.

Some of the American Nurses' Homes are wonderfully perfect, but certainly they are not all alike. A Superintendent there states: "I know at this writing of three hospitals in three different cities where the day nurses sleep in the beds occupied by the night nurses." We need make no comment on this unhygienic system, which we had ventured to hope was obsolete in America and in England. Male nurses have an opportunity in the United States which they cannot as yet obtain here, and we learn that twenty-seven young men have recently received diplomas on the completion of their two years' course at the Bellevue Hospital Training School for Male Nurses.

### SHORT ITEMS.

The new Nurses' Home at the Blackburn Infirmary will be completed by the end of this month. Mr. and Mrs. Henry Harrison are giving the whole of the furniture for it.—The Cottage Hospital at Brentwood has been closed, Mr. J. C. Goode, who was the chief contributor to its support, having left the neighbourhood. Surely the residents will not allow this useful little institution to cease to exist altogether.—At the annual meeting of subscribers to the Buckingham Nursing Home several entertainments given by friends in aid of the funds were reported. Dr. De'Ath's wish to be again one of the honorary medical officers of the home was cordially welcomed.—Miss Florence Moore received several handsome presents from the nursing staff on resigning her post of Night Superintendent at the Royal United Hospital, Bath.—The District Nursing Association at Newtownards, inaugurated under the auspices of Lady Londonderry, is likely to take shape very soon, as subscriptions are coming in satisfactorily. —Miss Lucas, of the North Cambs. Hospital, was presented with a purse of gold and other gifts on her resignation of the post of Matron.—A suitable Hospital for Women and Children is going to be built at Madras. The Government has sanctioned the outlay required for its erection.

### The Royal British Nurses' Association.

### THE FULL TEXT OF THE CHARTER.

VICTORIA, by the grace of God of the United Kingdom of Great Britain and Ireland Queen, Defender of the Faith, TO ALL TO WHOM THESE PRESENTS SHALL COME, GREETING.

Whereas it has been represented to Us by Our Most Dearly Beloved Daughter Helena, Princess Christian of Schleswig-Holstein, Princess of Great Britain and Ireland—

That in 1887 a Society was established in London called the "British Nurses' Association," which has since its establishment been joined by more than 3,000 Nurses, each one of whom has been engaged for three years or more in attendance upon the sick.

That the said Association was not established for the purpose of gain but for the purposes of the improvement of the profession of Nurses and of the promotion of their efficiency and usefulness and of assisting them by various benevolent schemes.

That in furtherance of the said objects a List of Nurses has been compiled and published setting forth the names and addresses of Nurses with the names of the Hospitals or Institutions at which they have been trained and the length of training which each has received thus enabling the public to form a more accurate judgment of the professional education and experience of the Nurses so registered.

That a benevolent fund has been established for the purpose of giving aid to Nurses, and that the management of the said Association is vested in certain eminent members of the Medical Profession, Matrons of Hospitals, and other persons interested in Nursing.

And that in 1891 We were graciously pleased to confer on the said Association the prefix and title of "Royal" and to command that the said Association should be styled the "Royal British Nurses' Association."

And Whereas Our said dearly beloved daughter is President of the said Association,

And Whereas it has been represented unto Us by Our said dearly beloved daughter that it would conduce to the welfare of the Association and to the furtherance of its objects of the said Association were Incorporated by Our Royal Charter,

And Whereas an application has been made to Us by Our said dearly beloved daughter so to incorporate the several persons hereinafter named and all other the members of the said Association,

And Whereas it appears to Us that a permanent Association formed for the purpose of maintaining a closer connection among persons practising as Nurses and thereby ensuring mutual counsel, comfort, and support, and for the purpose of disseminating to the public at large information respecting such persons is likely to prove of much public benefit, and that such purposes would be better ensured by the incorporation of such an Association.

Now know ye that We being desirous of promoting the said Association have of our especial grace certain knowledge and mere motion given and granted and We do hereby give and grant that

Our said most dearly beloved daughter Helena, Princess Christian of Schleswig-Holstein, Princess of Great Britain and Ireland, Our trusty and well beloved

Sir James Paget, Baronet.
Sir Spencer Wells, Baronet.
Sir William Savory, Baronet.
Sir Richard Quain, Baronet.
Sir Joseph Fayrer, Knight Commander of the Most Exalted Order of the Star of India.
Sir Henry Thompson, Knight Bachelor.
Sir James Crichton Browne, Knight Bachelor.
Sir Dyce Duckworth, Knight Bachelor.
Sir Edward Sieveking, Knight Bachelor.
Sir Alfred Garrod, Knight Bachelor.
Sir George Humphry, Knight Bachelor.
Robert Brudenell Carter, Fellow of the Royal College of Surgeons of England.
John Williams, Doctor of Medicine.
William Bezly Thorne, Doctor of Medicine.
Bedford Fenwick, Doctor of Medicine.
Isla Stewart (Matron and Superintendent of Nursing, of St. Bartholomew's Hospital).
G. M. Thorold (Lady Superintendent of the Middlesex Hospital).
Ethel Gordon Fenwick (late Matron and Superintendent of Nursing, of St. Bartholomew's Hospital).
Cassandra Beachcroft (Lady Superintendent of the Lincoln County Hospital).
Margaret Breay (Acting Matron of the Metropolitan Hospital).
Mary N. Cureton (Lady Superintendent of Addenbrooke's Hospital, Cambridge).
Christina Forrest (Lady Superintendent of the York County Hospital).
Louisa Hogg (Head Sister, Royal Naval Hospital, Haslar).
R. F. Lumsden (Hon. Lady Superintendent of the Royal Infirmary, Aberdeen).
Henrietta C. Poole (Nursing Superintendent, Adelaide Hospital, Dublin).
Gertrude A. Rogers (Lady Superintendent of the Leicester Infirmary).
Georgina Scott (Lady Superintendent of the Sussex County Hospital).
Maud G. Smith (Lady Superintendent of the Royal Infirmary, Bristol).
Catherine J. Wood (late Lady Superintendent of the Children's Hospital, Great Ormond Street).

And all other persons who pursuant to this Our Charter become members of the Corporation established by this Our Charter shall be a body corporate by the name of the Royal British Nurses' Association having a perpetual succession and a Common Seal with a capacity to sue and be sued in their Corporate name and for the purposes of the said Corporation to take, purchase and hold any personal property and also notwithstanding the Statutes of Mortmain any messuage land and hereditaments provided that the yearly value of such messuages, land and hereditaments shall not in the whole exceed £2,000, the yearly value of such messuages, lands and hereditaments being for that purpose taken to be the yearly value thereof at the times they are respectively acquired by the Corporation, With power to sell grant and demise mortgage or exchange and dispose of the said property messuages lands and hereditaments or any part thereof Provided that the Corporation shall apply its profits if any or other income solely in promoting its objects and for no other purpose.

And We do hereby declare as follows :—

*Preliminary.*

In the construction of this Our Charter the following words and expressions unless there is something in the context inconsistent with such interpretation shall have the meanings hereinafter attached to them ; that is to say—

"The Corporation" means the Royal British Nurses' Association, incorporated by this Our Charter.

"Person," includes a body of persons incorporated or unincorporated.

Words in the masculine gender include the feminine, and words in the singular number include the plural and in the plural number include the singular.

*Purposes and Powers of the Corporation.*

The purposes of the Corporation are the following :—

1. The founding and maintenance of schemes for the benefit of Nurses in the practice of their profession and in times of adversity sickness and old age.
2. The maintenance of an office or offices for supplying information to persons seeking for Nurses and to persons seeking for employment as Nurses.
3. The maintenance and publication of a list of persons who may have applied to the Corporation to have their names entered therein as Nurses, and whom the Corporation may think fit to enter therein from time to time, coupled with such information about each person so entered as to the Corporation may from time to time seem desirable.
4. The promotion of conferences, public meetings and lectures in connection with the general work of the Corporation.
5. The doing anything incidental or conducive to carrying into effect the foregoing purposes.

The Corporation may affiliate to themselves or amalgamate with themselves or enter into any arrangements for wholly or partially working in conjunction with any person or any body of persons corporate or unincorporate formed for all or any of the purposes for which the Corporation themselves are formed or for any purposes analogous or corresponding thereto and may for the purpose of carrying into effect this power contribute to or receive contributions from any person or from the funds of any such body of persons upon such conditions as to the corporation may seem fit or make such other financial arrangements from time to time with such persons or bodies as may be mutually agreed upon.

The Corporation may with a view of carrying into effect the purposes of their constitution or any of such purpose erect and maintain adequate buildings with such accommodation as may be deemed fitting for the purposes of the Corporation and may furnish the same with such requisites as may be necessary.

*Funds.*

All property now belonging to, or held in trust for, the Royal British Nurses' Association shall from the date of these presents vest in the Corporation.

*As to the President and Honorary Officers.*

There shall be a President of the Association and such number of Honorary Officers as the Association may from time to time think fit to appoint.

The first President shall be her Royal Highness Princess Christian of Schleswig-Holstein.

The first Honorary Officers shall be those persons who at the date of this Our Charter are Honorary Officers of the Royal British Nurses' Association in the recitals of this Our Charter specified.

On the resignation or death of the President or of any Honorary Officer and as occasion may arise the vacancies shall be filled up by the General Council hereinafter mentioned, upon the nomination of the Executive Committee hereinafter mentioned.

The President shall preside at all meetings of the General Council and Executive Committee or of any Sub-Committees thereof at which she may be present.

The President shall have power to summon meetings of the Corporation at any time she may think fit so to do, in order to submit to the Members thereof such matters of importance relating to the affairs of the Corporation as she may deem requisite.

*Members of the Corporation.*

The Members of the Corporation shall consist of the following persons—

1. The President.
2. All person who at the date of this Our Charter are members of the Royal British Nurses' Association in the recitals of this Our Charter specified.
3. Persons from time to time elected as hereinafter mentioned.
4. The Corporation may by Bye-law regulate the qualifications of candidates for election, but no such Bye-law shall come into force until approved by the Lords of the Privy Council

*General Meetings.*

There shall be a General Meeting of the Members of the Corporation within the space of six calendar months after the date of these presents. An Annual General Meeting of the Members of the Corporation shall thereafter be held once in every year at such time as may be prescribed by the Bye-laws for the time being in force, for the election of members of a General Council of Members of the Corporation and to receive Annual, Financial, and other Reports for the past year and for other purposes of the Corporation, and other general meetings may be held from time to time as occasion shall require and as the Bye-laws shall direct.

*Bye-Laws.*

At any General Meeting it shall be lawful for the Members of the Corporation or such of them as shall be then present to ordain and make such Bye-laws as to them or the major part of them shall seem proper for the regulation and good government of the Corporation and of the members and affairs thereof and generally for carrying the objects for which the Corporation is founded into full and complete effect and the said Bye-laws or any of them from time to time to alter change or annul as the said members in General Meeting shall think requisite so as all and singular such Bye-laws be reasonable and not repugnant or contrary to the provisions of these presents or to the Laws and Statutes of this Our realm, but no such Bye-law shall come into force until approved by the Lords of the Privy Council.

*General Council.*

The first General Council of the Corporation shall consist of the persons who at the date of Our Charter are Members of the General Council of the Royal British Nurses' Association in the recitals of this our Charter specified.

The General Council shall be the governing body of the Corporation and shall consist of such numbers of Members *ex officio* and elected Members possessing respectively such qualifications as may be prescribed by the Bye-laws for the time being in force. The Members of the General Council shall hold office for such period and retire therefrom in such rotation as may be prescribed by the Bye-laws for the time being in force. The General Council shall meet at such times as may be prescribed by the Bye-laws for the time being in force to receive the Report of the Executive Committee, and to transact such other business as may come before it. The ultimate decision on any matter affecting the Corporation shall rest with the General Council and the General Council shall have power to expel from the Corporation or suspend from membership any Member who shall after full inquiry and after hearing such Member be deemed by the Council unworthy to remain a member or to act as such by reason either of moral delinquency or professional incompetence provided always that no Member be so expelled or suspended at any meeting of the Council unless twenty-five Members of the Council at the least be present at such meeting and unless not less than three-fourths of those present and voting shall vote for such expulsion or suspension and provided further that any Member so expelled or suspended shall have the right to demand that before such expulsion or suspension takes place the matter shall be referred for decision to a General Meeting of the Members of the Corporation to be forthwith summoned for the purpose.

*The Executive Committee.*

The General Council shall annually elect to be Members of an Executive Committee such number of Members of the Corporation as may be prescribed by the Bye-laws for the time being in force.

The first Executive Committee shall consist of the persons who at the date of this Our Charter are members of the Executive Committee of the Royal British Nurses' Association in the recitals of this Our Charter specified.

The Executive Committee shall consist partly of members *ex-officio* and partly of elected members, in such numbers and proportions and possessing respectively such qualifications as may be prescribed by the bye-laws for the time being in force. The Members of the Executive Committee shall hold office for such period, and retire therefrom in such rotation, as may be prescribed by the Bye-laws for the time being in force.

The Executive Committee shall have power from time to time at their meetings to be held at the times and places to be prescribed by the Bye-laws of the Corporation to appoint and elect in the manner prescribed by such Bye-laws such persons as they shall think fit, being eligible under the provisions of these presents to be members of the Corporation.

The Executive Committee shall (subject to the powers herein vested in the General Meetings of the Corporation and in the General Council) have the sole and entire management of the business of the Corporation and of the income and property thereof for the uses purposes and benefit of the Corporation, and shall appoint such paid officers and servants and pay them such salaries or other remuneration as they may deem necessary or useful to the Corporation and may remove them if they shall think fit and shall prescribe their respective duties. The Executive Committee shall and may meet together when and as often as they shall think fit until the passing of the Bye-laws hereinbefore mentioned and from and after the passing of such Bye-laws at such times and places as shall be prescribed by the said Bye-laws. The Executive Committee may appoint such Sub-committees and delegate to them such duties as from time to time shall seem expedient, and the Executive Committee and such Sub-committees shall from time to time do all such acts as shall appear to them or to the majority of those then present necessary or fitting to be done in order to carry into full operation and effect the objects and purposes of the Corporation, so always that the same be not inconsistent with or repugnant to the provisions of this Our Charter, of any existing Bye-law agreed upon at any General

Meeting of the Members of the Corporation, or the Laws and Statutes of this Our Realm.

*Annual Report and Statement of Accounts.*

The accounts of the Corporation shall be audited annually by an auditor or auditors who shall be chartered accountants and shall be chosen at the Annual General Meeting in each year.

The Executive Committee shall once in every year at least prepare a General Report of their proceedings for the year preceding, and attach thereto a duly certified statement of accounts and of the finances of the Corporation.

Each Member of the Corporation shall pay such annual or other subscription as may be prescribed by the Bye-laws for the time being in force.

At every general or special Meeting of the Corporation every member of the Corporation shall have a vote, but no member shall be entitled to be present or to vote at such meeting who is in arrear of any subscription or other sum payable by him or her under the bye-laws for the time being in force.

At any such meeting and at any meeting of any Council Committee or Sub-Committee of the Corporation the person for the time being occupying the chair shall be entitled to vote, and shall also, in case the numbers voting upon any question shall appear to be equal on either side, be entitled to give a further or casting vote.

If any person ceases from any cause whatever to be a Member of the Corporation he or she shall not nor shall his or her representatives have any interest in or claim against the funds or property of the Corporation.

No act or thing done by the Corporation or by any Council Committee or meeting thereof shall be deemed to be invalidated by reason of the fact that a vacancy or vacancies may exist in any office or post appertaining to the said Corporation or in any Council or Committee of the Corporation at the time when such act or thing shall be done.

And lastly We do by these presents for Us and Our Royal Successors grant unto the said Corporation hereby established and their successors that these Our Letters Patent or the enrolment or exemplification thereof shall be in and by all things good firm valid sufficient and effectual in the law according to the true intent and meaning thereof and shall be taken construed and judged in the most favourable and beneficial sense for the best advantage of the said Corporation and their successors as well in all Our Courts of Record as elsewhere by all and singular Judges Justices Officers Ministers and other subjects whatsoever of Us Our heirs and successors, any non-recital mis-recital or any other omission imperfection defect matter cause or thing whatsoever to the contrary thereof in anywise notwithstanding.

In witness whereof We have caused these Our Letters to be made Patent.

Witness Ourself at Westminster, the sixth day of June, in the fifty-sixth year of Our reign.

By Warrant under the Queen's Sign Manual.

MUIR MACKENZIE.

### South Africa.

### ST. JOHN AMBULANCE ASSOCIATION.

Grahamstown Asylum.

The following members of the nursing staff passed the first examination of this association, held on December 12th, 1892, J. B. Greathead, M.B. Edin., M.R.C.S., examiner:—Males: Robert Walters, A. J. B. Botten, H. E. Wilson, J. Kinnell, W. H. Crampton, Geo. Elliott. Females: M. E. Parker, E. A. Comber, E. McCann, F. Robertson, C. Codd, A. F. Jeanes.

### District Nursing at Braintree.

For the last eleven years a most useful work has been carried on by the Braintree branch of St. Albans Diocesan Nursing Institution. Owing to the recent death of a lady who had almost entirely supported this branch of the institution, it will have to be closed, and the sick poor of the neighbourhood will thus be deprived of the services of the Sister-in-charge. Only those who have watched her work for years past can realise what a loss she will be to the neighbourhood.

### Surgical versus Medical Nursing.

By a Medical Nurse.

With the elevation of surgery to the front rank of scientific professions has arisen a demand for private nurses adapted by training and experience to surgical needs. In the home, as in the operating theatre, the skilled surgeon requires an assistant who, by alertness in anticipating his wants, and by the careful following out of his directions, will lessen the anxiety attending the performance of serious operations. This demand has been supplied by the nurse-training schools, which, by insisting on a long and complete term of instruction, have succeeded in producing attendants eminently adapted to the work.

Those who have been present at serious operations cannot have failed to notice the arrangement of the operating-room, the exquisite order and cleanliness secured by the hands of the nurse. Perhaps her accurate knowledge of the operator's wants struck us most. Hardly had the surgeon realised his need for either aid or instrument ere he found at his side the article or experienced hand he required. To such perfection has this part of nursing been brought that the results seem little short of miraculous to the unprofessional observer.

But this very perfection, admirable as it is, has another aspect, which has of late been much before that large portion of the public who need help in sick-rooms. It seems as if in selecting and training their probationers the hospitals have been too prone to keep chiefly in mind surgical needs. Perhaps the candidates have been principally selected for quickness of eye and hand, and general readiness in emergencies. Thus patience, sympathy, and unselfishness are not sufficiently sought for, or valued—those who possess these qualities do not always attain to manual dexterity. In looking at the report of the Nurses' Co-operation for last year we find that out of 1,732 cases attended by its members, only 412 were surgical, the greatest demand being made for medical nurses. This being the largest existing association for the supply of private nurses, we may take its report as a general guide to the class of attendants required.

By far the larger part of the community suffer from ordinary illness, surgical diseases being in the minority. Under these circumstances there may be some justification for the complaint, that too much attention is directed to the surgical part of a nurse's training, with the result that she is apt to regard medical work as less interesting and difficult.

The cause of the pre-eminence of surgical nursing is not far to seek; the glamour that surrounds the marvellous success of great surgeons is reflected on to their nurses, and thus it comes about that the work of the latter is more praised and better paid than other branches of the profession.

In any more ordinary case of illness we may, however, be surprised to find the large amount of work and of skill required from the nurse. Every hour, nay, sometimes every five minutes, attention is necessary, and she must be on the alert to minister to the many needs of her suffering patient. His recovery often depends greatly on wise nursing; weeks and months of watchfulness pass slowly by, calling for almost superhuman patience and for infinite tact. Anxious friends offer impracticable advice and suggestions as to treatment. The patient himself, worn out by continued illness, becomes depressed and irritable, and it requires all the nurse's devotion to enable her to bear the constant demands made upon her. In this class of work there is not the interest attendant on daily dressings, &c. Yet the woman who has successfully nursed a bad case of typhoid or hæmoptysis should, from the nature of her responsibilities, certainly rank as high as one who has tended a serious operation case. The latter requires mechanical quickness and acquaintance with certain traits, which can be attained by practice, but the former needs a greater knowledge than this, or she will be of little use to the medical man, obliged to rely on the accuracy of her observation. A conscientious discharge of her duties should characterise every nurse, and in praising the surgical it is not necessary to disparage her medical sister.

### The R.B.N.A. Charter.

We have received the following communication from a nurse on this subject:—

Royal Charters share the characteristic of other legal documents and employ a redundancy of expression which tends to obscure their objects. On the minds of ordinary people, such as ourselves, this elaborate phraseology has a rather bewildering effect, but as we nurses have, at least, average intelligence (otherwise how should we have got through the examinations included in our training?) we may as well understand how much and how little the charter really means. The kernel of the nut is contained in the statement in the charter of the purposes for which it has been granted, and a very satisfactory kernel it is. It settles all the doubts which have spread and multiplied respecting registration, compulsory or otherwise. The charter does not establish a register, that is the chief thing which concerns us as nurses. A general public register was, on very sufficient grounds, protested against by Miss Florence Nightingale and the heads of many of the London and provincial training schools, and therefore it was improbable that the Queen, who has proved herself so true a friend to nurses, would act in opposition to the wishes of their teachers.

### Where to Go.

The opening of the Nurses' Home at Derbyshire Royal Infirmary is announced for the 29th inst. Tea and music will be provided in the hospital grounds, from four to six p.m., by Miss Wilmot, the Mayoress of Derby, and other ladies.

A lecture will be given on June 23rd, at 8 p.m., by Mrs. Schailieb, M.D., B.Sc., at the Trained Nurses' Club, 12, Buckingham Street, Strand. Subject: "Hæmorrhage before Parturition." Non-members' tickets, 6d. each.

Free organ recitals at the Albert Hall, commencing at three o'clock, on Sunday afternoons.

The Prince and Princess of Wales will open a bazaar in aid of the funds of the Alexandra Hospital for Children with Hip Disease, Queen Square, W.C., to be held at the Westminster Town Hall, on the 3rd, 4th, and 5th of July. Their Royal Highnesses will be present at one o'clock on the 3rd of July. Stalls will be held by many well-known leaders in society, and the band of the Royal Artillery will be present.

National Workmen's Exhibition at the Agricultural Hall.—We understand that an interesting feature of this exhibition, which is to be opened by the Prince of Wales on July 1st, will be a stall for the display of homespuns, hosiery, Irish lace, and other productions of the various industries promoted and encouraged by the Donegal Industrial Fund under the management and direction of Mrs. Ernest Hart. The Lady Mayoress has, in the kindest manner, promised to preside at this stall on the opening day, in the absence of Mrs. Hart, who is at Chicago superintending her Irish Village in the World's Fair.

### Disinfection in Spain.

By Royal order the Governor of a Spanish town can take all steps which he thinks necessary in combating an epidemic. He has authority to condemn houses and to burn the effects of the occupiers. If the persons thus obliged to move and to lose their clothes, &c., are poor, they receive relief, and are indemnified for all losses at the cost of the Government. The Town Council provides disinfectants, and the loan of a stove free of cost whenever requested to do so.

### Our Examination Questions.

Our June question has received so many answers that it is evident the prevention of bedsores comes within the range of most persons' knowledge. Suggestions of careful, patient nursing appear in many of the papers, a large proportion of these, by the way, being exceptionally good. Some writers speak from a more limited experience than others, but they have all learnt what to avoid if they wish their patients to keep free of pressure sores.

Probably infirmary nurses have the most extensive knowledge of the worst kind of these, for their charges have often acquired terrible ones through lack of care and cleanliness at home, and the same may be said regarding the experience of nurses in hospitals for chronic diseases. In general hospitals "prevention" is the part of the subject most familiar to the nursing staff, and the latter is apt to speak with much scorn of the disgrace which the development of a bedsore on one of her patients would bring on a nurse. A short residence in a workhouse or similar institution, however, teaches that the "cure," difficult and tedious as that may be, will be as frequently her duty as the prevention. In the majority of answers sent us we are glad to notice that much stress is laid on frequent washing of the skin with plenty of soap and water, only a few competitors speaking of spirit and powder as substitutes, instead of additions to, the washing. The combination of oil and spirit, which suits so many skins, does not seem in very general favour, and a great deal too much importance is attached to powders. These are useful enough in moderation, but distinctly harmful in large quantities, and they "cake" objectionably when used in connection with the greasy applications to which some nurses seem unduly devoted.

We are pleased to find it generally conceded that the doctor ought to be told of the first indication of a bedsore, and only a few nurses venture to lay down arbitrary rules of treatment without reference to the medical man's orders. We give the prize answer below.

Examination Question for June.

What are a nurse's duties with regard to the prevention and treatment of bed sores?

Answer.

A bedsore is produced by pressure on prominences of the body, such as the heels, shoulder blades, elbows, &c., but most commonly over the sacrum. Three main points in prevention are, removal of pressure from the part threatened, cleanliness, complete dryness of the bed. Some doctors hold that sores are always preventable, but in a few cases it seems impossible to avoid them. The skin of any part likely to become broken should be well washed twice a day with warm water and plenty of soap, after which it should be thoroughly dried and rubbed with brandy, methylated spirit, camphorated spirit, or some other hardening application. Boracic ointment is sometimes more effectual than anything else in warding off bedsores. After the lotion or ointment has been well rubbed in, the place may be dusted with starch and zinc powder. Some nurses paint the tender part with collodion, or brandy and white of egg, but neither is much to be recommended. Great attention must be paid to dryness of the bed; it will be impossible to avoid sores if the patient be left lying on a wet sheet. Smoothness of the bedclothes is another essential; any seams or rucks in sheet, mackintosh, or under-blanket will produce bad results and great discomfort to the patient. Care must be used in giving and taking away the pan, as with helpless patients it is liable to injure the skin over the sacrum. Change of position is an important aid in preventing sores. The patient should be moved if possible from side to side, alternating this with lying on his back. When this cannot be done, raise or lower the part threatened by putting in or taking away a pillow. Where the back is the affected place an air or water ring may be of use. If a bedsore seems imminent, the fact must be reported to the doctor at once, and his directions as to treatment carefully carried out. Should it be a case where the nurse has to act on her own responsibility, she may apply some ointment on lint, covered by a large pad of cotton wool or gamgee, held in its place by strapping or bandages; this can be changed as often as necessary. She should continue to wash daily the parts round the sore, avoiding the broken place, and applying the lotion or ointment as before. In some cases bathing the sore with red lotion will be found stimulating. A water bed will often be found invaluable. All crumbs must be at once swept out of the bed, and anything else of a similar nature that could hurt the patient's skin.—A. Jelf. [We have had a letter which we sent to Miss Jelf returned as "insufficiently addressed." If she will write again we shall be pleased to communicate with her about the prize.]

*** An excellent article on Bedsores appeared in The Hospital of January 28th, 1893, page cxxxi.—Ed.

Examination Question for July.

(*a*) Describe carefully the different ways of making a bed with a patient in it. (*b*) The precautions to be taken when a patient gets up whilst the bed is made.

Answers must be sent in by July 12th. They must be written on one side the paper only, and accompanied by writer's name and address, and directed to "Nursing," Editor of The Hospital.

### Everybody's Opinion.

[*Correspondence on all subjects is invited, but we cannot in any way be responsible for the opinions expressed by our correspondents. No communications can be entertained if the name and address of the correspondent is not given, or unless one side of the paper only be written on.*]

### THE ROYAL WEDDING.

"An Infirmary Nurse" writes: Please allow me through your columns to ask if it could be arranged for matrons, sisters, and nurses to have a stand to themselves, so that they might comfortably view the Royal procession. I belong to the Mary Adelaide Nursing Association, and should like it to be well represented on the occasion.

### A PAYING COTTAGE HOSPITAL.

"Sussex Matron" writes: A partly-paying cottage hospital in Sussex has just closed its wards (six beds) for want of funds. I shall be pleased to furnish "E. M. N." with particulars of the management and of the need for such a place. It has been doing very good work. If funds were forthcoming the hospital might be made a great success, for it is in a town. Will "E. M. N." kindly communicate with "Sussex Matron," The Hospital, 428, Strand.

### Hospital Discipline.

Last Saturday afternoon a fire broke out at Messrs. Cubitt's premises in Gray's Inn Road. As the buildings are close to the Royal Free Hospital it was feared lest this also should be attacked by the flames, and the patients were promptly removed from the threatened wards by the medical staff, nurses, and students, who acted with promptitude and method. There was no confusion, and no harm has resulted to any of the patients. Regular fire drill has for some time systematically taken place at the hospital under the instruction of an officer of the fire brigade. Whilst glad to record that the hospital escaped any damage, we congratulate the staff on their admirable presence of mind.

### Minor Appointments.

Victoria Hospital, Burnley.—Miss Bullock has been appointed Sister of the children's ward at the Victoria Hospital, Burnley. Miss Bullock was trained at the Royal Southern Hospital, Liverpool.

### For Reading to the Sick.

### LOVE.

Love is the most attractive quality of the Godhead, and the writers of both the Old and New Testaments are constantly drawing our attention to the fact that God is Love. Yet people too often think only of His judgments and His punishment of the wicked, and say He is a jealous God, visiting the sins of the fathers upon the children; and while dwelling on this point they forget the loving messages He was constantly sending to His rebellious children the Jews in order to allure them back to their allegiance; and the equally loving words He bestows on us Christians. It was necessary at first to send the law in terrors to teach men the extreme sinfulness of sin; but

When God came the second time
He came in power and love:
Softer than gale at morning prime
Hovered the Holy Dove over the man Christ Jesus, the meek, the gentle healer of all who were sick and sorrowing. It is He who has taught us to pray to the Almighty as Our Father, a name of fondest affection which we share with our blessed Lord Himself.

We often make mistakes about things in our lives, but we can never be mistaken in saying that God's love is *perfectly certain*. We are quite *sure* He loves everybody, and would have all men to be saved. The King and the beggar, the rich and the poor, the aged and the infant, the wise and the ignorant, the sick and the whole, the strong and the weak, He loves them all as truly as He made them all. It has been said, "We may shut out the light of day from our houses, but we cannot shut out the light of God's love. We may weary our friends with importunate cravings, but we cannot weary God's love; we may lose all that is worth possessing on earth, but we can never lose God's love." By day and by night, in sleeping or waking, we are His care, for He that watcheth over Israel shall neither slumber nor sleep.

God is so patient, too, though we provoke Him daily without even recognising how wrong we are, yet He endures it, hoping that we shall amend. If there is anything which should bring down our pride and make us think humbly of ourselves, it is the love of God. And what have we done to deserve it? Nothing; we have not a single claim on Him except His great love for us, and He asks nothing from us except *our* love. "My son," He says, "Give Me thine heart." The great commandment of the law is, thou shalt love the Lord thy God with all thy heart. Let us earnestly try to keep it, because obedience is easy where there is love; we shall then no longer think of terrors and judgments, for perfect love casteth out fear.

### A Sad Tragedy.

A terrible accident occurred at Bengeo College, Hertford, last week, a schoolboy of twelve years old being poisoned by a dose of carbolic acid given instead of cough mixture. That the bottle was in the wrong place and the nurse too sleepy to know what she was doing can give little comfort to the distressed parents. As there was another nurse in the house it seems strange that Nurse Barnard should have been in bed in the room with five patients, and that when the poor little lad's cough disturbed her and she got up to attend to him she was not properly awake. Night nursing needs even more alertness than day duty, and during the latter a nurse would not be found in bed. There appears to have been defective management somewhere, and unfortunately nothing can undo the disastrous results. Parents and schoolmaster must alike command our warmest sympathy.

### A Groat's Worth of Wit.

It was one of those days last month that tasted like midsummer, and late in the afternoon a boat was gliding lazily down stream between Streatley and Pangbourne. The rowers rested on their oars to listen to the first notes of the cuckoo that day arrived, and the stillness was broken by no other sound but the splash of some water-rats swimming close in shore. The sun was nearing the resting place of the long, low hill, and the right bank lay half in shadow, while on the left the fields were lighted up in all their spring splendour of brilliant green and yellow. There were cows munching monotonously, and a lazy collie—the same who had frightened the water-rats—was sitting on his haunches on the towing-path. Overhead a clear blue sky; whiffs of lilac and hawthorn were about, and in the whole atmosphere the curious, contented feeling of placid laziness which haunts those upper reaches of the Thames.

And the centre of this little picture, all unconsciously to herself, was a hospital nurse. She was standing by a gate in regulation dress, with a black veil streaming behind, and to those who caught a passing glimpse as they floated by, it seemed as though all the harmony of mingled sight and sound and scent had been arranged to rest the tired brain and whisper to her the glad message of spring, that the forces of life are stronger than the forces of decay, and that beauty and love are triumphant even in the midst of death itself.

Was she surrendering herself to the spirit of the hour, and drinking in fresh hope and vigour with store of peaceful thoughts which should turn to peaceful memories? I hope so; but I doubt. In the first place, she was standing erect and resolute, with one hand resting on the gate. Now the proper attitude of enjoyment for a woman is to lean, or to sit. There was accommodation for either, but she chose to stand. And then from the glimpse of the face to be had in passing, it was clear that the muscles were not relaxed, there was tension in the knit brow, the lips were firmly compressed; the very chin was anxiously poised. I could not see the eyes; they were looking into space, and evidently saw nothing of the fair scene before them. In my opinion, they saw bandages, medicine bottles, and beds. She was thinking of her case. I take it she had come off duty for an hour or so from a private patient. Now what would a man have done under the circumstances? He would have chosen by instinct the softest spot of turf, he would have stretched himself out entirely at ease, he would have lighted his pipe and in his widest range of thought he would never even distantly have approached the object of his working hours. Which of the two would have profited most?

Now to speak frankly this seems to be one main cause of woman's inferiority (even the most superior will admit perhaps that some of us are inferior). A woman will not leave her work alone. She will not take the trouble of acquiring the art of relaxation. Perhaps it comes from her laudable and abnormal appetite for work. All women at heart are like the sexton who spent his one holiday in twenty years inspecting a neighbouring cemetery. Even in her one long holiday in the year the woman worker finds it very hard to dismiss "shop." If she is a teacher she will be storing her mind with illustrations. If she is a nurse she will be talking "hospital" on the slighest provocation, or without it; she will be sitting up all night with her landlady and prescribing for the bathing woman's twins. Still most people—even women—can take a rest if they give their minds to it for a fortnight together.

But where the women fail is in their odd moments. They are hard to please, in more senses than the poet meant, in those off duty times which ought to be, but are not, hours of ease. They carry the worries of the ward with them into the street and revolve the impracticability of the sister, or the crass stupidity of the lady probationer, in a brain which goes on turning in a vicious circle till the body is jaded to match the mind.

A kind lady travelled not long ago all the way from America to teach the British woman the art of resting. She gave lectures on the right way to sit and lie, and she tried to impart the art of unstringing the mental bow and letting all the muscles relax with it in moments of repose. It seems to be one of those lessons, like experience, which have to be learned and cannot be taught. Look round at the women you meet in an omnibus or train. You will hardly see one face expressive of a mind at ease and prepared to enjoy. They are anticipating some coming interview, writing some difficult letter, or recalling some annoying blunder of yesterday. And they are all mentally pushing the vehicle on with every nerve at work; taking far more out of their whole sytem than if they were steadily employed in the regular daily routine.

But if the mind were at ease, even an omnibus journey might be turned to recreation. Many and various are the picturesque glimpses to be had in the London streets, and it is the eye that habitually fails to discover them which will be inattentive also to the charms of the riverside in spring, or will fasten on a novel among the glories of the Alps.

Too often the poet's voice is unheeded when he sings that

> . . . . "pleasure is spread through the earth,
> In stray gifts to be claimed by whoever shall find."

The gift is passed heedlessly by, perhaps as too insignificant, or perhaps as needlessly distracting, from cares which seem at the moment all important. Yet is anything insignificant which raises the mind for a moment "above the smoke and stir of this dim spot?" And can any restless revolving of cares be compared in importance with that habit of serenity to be attained by rising above them? Such serenity is in the reach of all, in whatever situation of life. It is the special need of those who are called by duty to association with every form of restless agitation. It can be won by constant efforts to rise at every opportunity, be it only a five minutes' pause while waiting for orders, out of the dull worries of every day life to a perception of the eternal beauty manifested all round us in little things. So that to the collected soul the busiest day shall be full of resting points, which, like tiny dewdrops, sink into the heart upraised to receive them, and reveal their healing power in an abiding freshness and increasing strength.

K.

### Notes and Queries.

### SPECIAL NOTICE.

The contents of the Editor's Letter-box have now reached such unwieldy proportions that it has become necessary to establish a hard and fast rule regarding Answers to Correspondents. In future, all questions requiring replies will continue to be answered in this column without any fee. If an answer is required by letter, a fee of half-a-crown must be enclosed with the note containing the enquiry. We are always pleased to help our numerous correspondents to the fullest extent, and we can trust them to sympathise in the overwhelming amount of writing which makes the new rules a necessity.

### Queries.

(152) *Training.*—Where can I get a certificate for one year's training?—*N. Richards.*

(153) *Poultices.*—Will you inform me how a poultice should be made and a fomentation applied?—*Attendant.*

### Answers.

(152) *Training.* (*N. Richards*).—Get "How to become a Nurse," by Honnor Morten, price 2s. 6d, at Scientific Press, 428, Strand.

(153) *Poultices.* (*Attendant*).—Under "Our Examination Questions," a full description of these was given in The Hospital of April 15th.

### Wants and Workers.

*A. C. L.* will be glad of information as to missions and nursing of British lepers.

*Sister Katherine, St. Mary's Home, Plaistow,* gratefully acknowledges a gift of nursing books, from Mr. Lewis, for her nurses' library.

### The Book World for Women and Nurses.

[We invite Correspondence, Criticism, Enquiries, and Notes on Books likely to interest Women and Nurses. Address, Editor, THE HOSPITAL (Nurses' Book World), 428, Strand, W.C.]

THE HYGIENE OF BEAUTY. By Dr. E. MONIN (of Paris), Secretary of the French Society of Hygiene; Chevalier of the Legion of Honour; Officer of Public Instruction. Translated under the immediate supervision of the author. Seventh edition, revised and considerably enlarged. (More than 200 recipes for medicinal and cosmetic preparations). (London: Baillière, Tindall, and Cox, 20 and 21, King William Street, Strand. Paris and Madrid).

This book will probably appeal to a larger, but perhaps less wise audience than its author intended. Dr. Monin is a hygienist, not a quack, and while he is fully alive to the importance of beauty he makes it go hand in hand with health. His chapter on paints is one of the briefest and least sympathetic in the book, and that on cosmetics deals with nothing more harmful than soaps. Moreover, much of his advice shows what to avoid as well as what to use. He quotes with approval Von Frenchtersleben's dictum: "Health is beauty in the functions of life"; his own teaching might be stated as the converse: "Beauty is health in the functions of life." The prescriptions given at the end of the book seem to be all harmless, and those which we have tried have proved in every way satisfactory. The advice as to baths and exercise is thoroughly sound, though perhaps it is more needed on the other side of the Channel than here. One might perhaps take exception to the recommendation of dancing as an exercise for young girls, not because it is unhealthy in itself, but it is often carried on in an impure atmosphere. This is a book which may legitimately find a place in every lady's library, nor need she blush if husband or lover finds it there.

A BABY'S REQUIREMENTS: By ELISABETH ROBINSON SCOVIL, Superintendent of Newport Hospital, R.I. (Curtis Publishing Company, Philadelphia. Price, 25 cents.)

Miss Robinson Scovil states in the preface of her little book that she has written it in compliance with the requests made to her in over twelve hundred letters. This is strong evidence of her being looked upon as an authority on a subject of much importance. That babies should have the best possible chance given them of developing into healthy men and women we must all agree, and many young mothers will be helped in the accomplishment of this end by Miss Scovil's practical hints. She seems to have remembered every detail which appertains to the comfort of an infant, and to have treated each with excellent common sense. Her suggestions as to the judicious care which women should take of their own health are as good as her detailed directions for the management of infants themselves. Diet, washing, amount of sleep, &c., required by babies are adequately described, and recipes for simple and suitable foods are given. "A baby's stomach requires rest just as its other organs do. To feed it every time it cries is as absurd as it would be to poke food down the throats of grown persons every time they yawned or groaned. Many lives are sacrificed to over-feeding," says Miss Scovil, very truly. The outfit necessary for a baby is admirably set down, the prices of materials, convenient patterns, and the number of garments which should be provided, being all carefully chronicled. Of course, all readers may not agree with the figures quoted, but these are naturally, to a certain extent, governed by the laundry arrangements of each individual household. Most of the baby clothes described are pretty, without being extravagant, but we do not understand why an infant of four months old should be put into long black stockings. Everything else being white, there are obvious reasons against black of any kind being introduced into an infant's wardrobe. However, this is a suggestion which need not necessarily be followed, and most of the hints are worthy of attention. The baby's basket and cot and the mother's bed receive due attention, and altogether "A Baby's Requirements" can be cordially recommended to nurses and mothers.

DOMESTIC MEDICINE AND HYGIENE: Being a short account of the more Common Diseases, their Causes and Treatment, written in plain language. By WM. J. RUSSELL, M.B. Third Edition. (London: W. H. Everett and Son.)

There is ample room for a wide difference of opinion as to the utility of such works as this. They are capable of imparting a great deal of most useful information, but they are also capable to fostering an over-weening self-confidence. Granting this position, the little book before us is good of its kind. The wording is everywhere simple and direct, and the directions seem plain and trustworthy. Some of the diseases included are rather difficult for the unscientific reader to recognize, *e.g.*, labio-glassal paralysis; adema-glottidis. The advice to an inexperienced layman to do laryngotomy is at any rate not lacking in courage; to paint the larynx with a solution of nitrate of silver, again, is not an easy task to accomplish. Other little details might be mentioned, but only encumber a book written for the public, but allowing for this the little manual is well done.

### NEW PERIODICALS.

THE LADY'S WORLD (Offices: 5 and 6, Henrietta Street, Covent Garden. Price 3d.).—The *Lady's World* is amongst the latest of a host of periodicals which have appeared since the New Year. Most of the better class ladies' weekly journals are published at sixpence, rather a large sum for slender purses, and if the *Lady's World* can keep up a fair standard of excellence, no doubt an amount of patronage will be accorded to it. Paper and printing are both good. The woodcuts are excellent, and they are used judiciously to illustrate the letterpress. The frontispiece at present is devoted to portraits of lady artists. The news is well up to date, and the various articles are pleasingly written and well chosen as to subject. "Dress at the National Gallery" will have interest for many, and may offer suggestions for the vexed question of "something new" for fancy balls. We are glad to see that sports for women are taken up in a businesslike manner. The garden, amusements, furnishing, fashion, and, in short, all subjects likely to suit the varied tastes of lady readers, seem provided for. A large space in No. 2, which we have before us, is devoted to charity, and the work at St. Mary's Day Nursery at Plaistow is most fully shown. The picture of the swinging cots is very fascinating, and we only hope they are as safe as they are pretty. Finally, there is the "Junior World" as supplement, in which, we are glad to see, that as yet no little nursery prodigies are perpetuated through the medium of objectionable rows of portraits.

